# What does music express? Basic emotions and beyond

**DOI:** 10.3389/fpsyg.2013.00596

**Published:** 2013-09-06

**Authors:** Patrik N. Juslin

**Affiliations:** Department of Psychology, Uppsala UniversityUppsala, Sweden

**Keywords:** music, emotion, expression, communication, categories, dimensions

## Abstract

Numerous studies have investigated whether music can reliably convey emotions to listeners, and—if so—what musical parameters might carry this information. Far less attention has been devoted to the actual *contents* of the communicative process. The goal of this article is thus to consider what types of emotional content are possible to convey in music. I will argue that the content is mainly constrained by the type of coding involved, and that distinct types of content are related to different types of coding. Based on these premises, I suggest a conceptualization in terms of “multiple layers” of musical expression of emotions. The “core” layer is constituted by iconically-coded *basic emotions*. I attempt to clarify the meaning of this concept, dispel the myths that surround it, and provide examples of how it can be heuristic in explaining findings in this domain. However, I also propose that this “core” layer may be extended, qualified, and even modified by additional layers of expression that involve intrinsic and associative coding. These layers enable listeners to perceive more complex emotions—though the expressions are less cross-culturally invariant and more dependent on the social context and/or the individual listener. This multiple-layer conceptualization of expression in music can help to explain both similarities and differences between vocal and musical expression of emotions.

## Introduction

Few scholars would dispute that music is often heard as expressive of emotions by listeners. Indeed, emotional expression has been regarded as one of the most important criteria for the aesthetic value of music (Juslin, 2013). Music has even been described as a “language of the emotions” by some authors (Cooke, 1959). It is not surprising, then, that a number of studies have investigated whether music can reliably convey emotions to listeners, and—if so—what musical features may carry this information. Far less attention has been devoted to the actual *contents* of the communicative process. The goal of this article is thus to take a closer look at the emotional contents of music. To be clear, the focus is on the *expression* and *perception* of emotions, rather than on the *arousal* of emotions (Gabrielsson, 2002).

In one sense, the term “emotional expression” is slightly misleading: it is only sometimes that musicians are truly expressing their own emotions in a composition or performance. What is usually meant by the term emotional expression is that listeners perceive *emotional meaning* in music. Yet the term “emotional expression” is widely established and will thus be retained in the present essay. The fact that people like to use the term “expression” suggests that music somehow reminds them of the ways humans express their states of mind in real life—a notion that is not too far off the mark (see section Iconic Coding: Basic Emotions).

Whereas Budd (1985) defined music as “the art of uninterpreted sounds” (p. ix), the present author instead assumes that music is *constantly interpreted*. Sometimes these interpretations may lead to the *arousal* of an emotion (e.g., Juslin, 2013). But more commonly, perhaps, we merely detect meaningful information. The notion of meaning suggests that music somehow *refers* to something else, beyond itself (Cross and Tolbert, 2009), but what kind of meaning it conveys has been a matter of much debate. Throughout history, music has been regarded as expressive of motion, tension, human characters, identity, beauty, religious faith, and social conditions. However, the most common hypothesis is arguably that listeners perceive music as expressive of emotions (for a review, see Gabrielsson and Juslin, 2003).

Empirical research largely confirms this view; for example, in a survey study by Juslin and Laukka (2004), 141 participants were asked what, if anything, music expresses. They were required to tick items from a list of options, based on a thorough survey of the literature on expression in music. Results indicated that “emotions,” unlike any of the other options, was selected by 100% of the participants. The real puzzle, however, and the topic of the present discussion is this: which emotions are expressed in music and why? The previous literature presents a somewhat confusing picture: some authors write about “expression” as something vague and flexible, almost idiosyncratic; others seem to view expression as something more specific, something for which terms like *agreement* and *accuracy* seem applicable. Are they really writing about the same phenomenon? It is hoped that the present essay can bring some clarity to this issue and illustrate how different conceptions of expression might be related.

The rest of this article is organized as follows. First, I briefly review some evidence regarding what emotions music typically express. I also discuss which approach to emotion—categories or dimensions—can best account for these results. Then, I argue that the emotional content of music is constrained by three types of coding that can be conceptualized as distinct “layers” of musical expression. Finally, I consider the implications of this conceptualization for the field.

## Which emotions does music express?

Note that there are different senses in which music can be said to express emotions. Firstly, a listener could perceive *any* emotion in a piece of music; and in a nontrivial sense, it would be inappropriate to claim that the listener is “wrong.” The subjective impression of an individual listener cannot be disputed on objective grounds. A first way to index emotional expression is thus to accept the *unique impressions* of individual listeners: Whatever a listener perceives in the music *is* what the music is expressing—for him or her at least! This is the view adopted by MacDonald et al. (2012), when they note that “we are … free to interpret what we hear in an infinite number of ways” (p. 5).

Several researchers prefer a more “restrictive” view on expression, however, which holds that music is expressive of a specific emotion only to the extent that there is some minimum level of *agreement* among different listeners regarding the expression, presumably because there is something in the music that produces a similar impression in many listeners. Expression thus conceived brings a stronger focus on psychophysical relationships between musical features and perceptual impressions. Thus, a second way to index emotional expression in music is to focus on listener agreement (Campbell, 1942).

The notion of expression does not require that there is any correspondence between what the listener perceives in a piece of music and what the composer or performer intends to express. In contrast, the concept of “communication” requires that there is both an intention to express a specific emotion and recognition of this emotion by listeners. Presumably, many musicians care about whether listeners perceive their music the way they intended it. Hence, if we study expressed emotions in terms of communication, we might also index emotional expression in terms of *accuracy* (Juslin and Timmers, 2010; see, e.g., Thompson and Robitaille, 1992).

Most likely, there are fewer emotions for which there is *agreement* among several listeners than there are emotions that a *single* listener may perceive in a piece. Even fewer emotions may be relevant if we consider those emotions that might be reliably *communicated* from a musician to a listener; that is, where there is an intention to convey an emotional character, which is correctly recognized by a perceiver. Later in this essay, I will offer a conceptualization that covers all of the above ways in which music could be said to express emotions—from the most personal to the most communal aspects of perceived expression.

Just as there are many different ways to conceptualize expression in music, there are different approaches that may be adopted to investigate empirically which emotions music can express. One rather simple way to approach the question is to ask music listeners directly. Thus, Table 1 shows data from three different studies in which listeners were asked which emotions music can express. In each study, the subject could choose from a long list of emotion labels. Shown are the rank orders with which each of the top ten emotion terms was selected. As can be seen, *happiness, sadness, anger, fear* and *love, tenderness* were all among the top-ten emotions, and this tendency was similar across the three data sets, despite differences in samples (musicians vs. students, various countries) and selections of emotion terms (ranging from 32 to 38 terms). Hence, there seems to be agreement about which emotions are easiest to express in music^1^

**Table 1:** **Ratings of the extent to which specific emotions can be expressed in music**.

	**Kreutz (2000)**	**Lindström et al. (2003)**	**Juslin and Laukka (2004)**
**Subjects**	**50 students**	**135 expert musicians**	**141 volunteers**
**No. of emotions Subjects**	**32**	**38**	**38**
**RANK ORDERING**			
1.	**Happiness**	**Joy**	**Joy**
2.	**Sadness**	**Sadness**	**Sadness**
3.	Desire	**Anxiety**	**Love**
4.	Pain	**Love**	Calm
5.	Unrest	Calm	**Anger**
6.	**Anger**	Tension	**Tenderness**
7.	**Love**	Humour	Longing
8.	Loneliness	Pain	Solemnity
9.	**Fear**	**Tenderness**	**Anxiety**
10.	Despair	**Anger**	Hate

Only the ten most highly rated emotions in each study have been included in the Table. Those emotion categories that correspond to the basic emotions are set in bold text. (Anxiety belongs to the “fear family,” and tenderness to the “love” family, see, e.g., Shaver et al., 1987.) The original lists of emotion terms contained both “basic” and “complex” emotions, as well as some terms commonly emphasized in musical contexts (e.g., solemnity).

It could be argued that such findings are more reflective of the beliefs and folk theories that musicians and listeners have about music than they are of any real circumstances. However, evidence that there is some substance to their intuitions comes from studies, where listeners are asked to rate the emotional expression of actual pieces of music. The results from over a hundred studies demonstrate that music listeners are generally consistent in their judgments of expression. Thus, a second approach to answer what emotions music expresses is to look at what emotions tend to yield the highest levels of agreement between listeners in previous studies. For instance, in their respective overviews, Gabrielsson and Juslin (2003) and Juslin and Laukka (2003) noted that the highest agreement between listeners occurred for emotions such as *happiness, sadness, anger*, and *tenderness*, and emotion dimensions, such as *arousal*. Moreover, there was often good agreement regarding the *broad* emotional character, but less agreement about *nuances* or *variants* of this emotion. Low agreement was found for emotion labels such as *jealousy*, *pity*, *cruelty*, *eroticism*, *whimsiness*, and *devotion*. In addition, hardly any agreement at all was found for various events depicted in so-called “program music.”

In sum, previous research suggests that certain emotions are easier to express in music than others. What approach can best help to explain these findings? To answer this question, we first have to review major approaches to conceptualizing emotions.

## Does musical expression involve categories or dimensions?

The dominant approaches to conceptualizing emotions in psychology are *categorical* and *dimensional* approaches, respectively. (I prefer to refer to them as approaches, rather than theories, because they represent broad perspectives on similarities and differences among emotions, which may include quite different emotion theories of a more specific kind.)

### Categories

According to categorical theories, people experience emotional episodes as categories that are distinct from each other, such as *happiness*, *sadness, anger, surprise, fear*, and *interest* (Izard, 1977). Note that categorical theories of emotion come in many different forms. Thus, one type of theory is associated with the concept of basic emotions (see section The Concept of Basic Emotions). However, many other emotion theories, such as component-process theories and “music-specific” models, also involve categories and therefore represent subdivisions of the categorical approach rather than additional approaches. Component-process theories (e.g., Scherer, 1984) assume that there are as many categories as there are possible outcomes of the appraisal process. A “music-specific” model assumes that the categories are different from “everyday emotions,” and, moreover, that they are “unique” to music. Zentner et al. (2008) proposed nine categories.

### Dimensions

In contrast, dimensional theories seek to conceptualize emotions based on their approximate placement along broad and continuous dimensions, such as *valence, activation*, and *potency*. Just like categorical approaches, dimensional models come in several different forms—from one-dimensional *arousal* models (Duffy, 1941), to two-dimensional (e.g., *Arousal-Pleasure*, Russell, 1980; *Positive Affect-Negative Affect*; Watson and Tellegen, 1985; *Energetic Arousal-Tense Arousal*; Thayer, 1989), or three-dimensional models (*Energy Arousal-Tense Arousal-Valence;* Schimmack and Grob, 2000; *Gaiety-Gloom*, *Tension-Relaxation*, *Solemnity-Triviality*; Wedin, 1972). The most popular version is clearly the *circumplex model* outlined by Russell (1980), maybe because it is easy to understand. It consists of a two-dimensional and circular structure featuring the dimensions *pleasure* and *arousal*. The model illustrates that emotions vary in their degree of similarity, and that some emotions are usually thought of as opposites.

### Discriminating empirical evidence

Which of these approaches best accounts for emotions? Many researchers view categorical and dimensional approaches as “complementary” (Nyklíček et al., 1997): they both receive some support from neurophysiological findings (Damasio, 1994), and both can be useful to characterize emotions in music (e.g., Vieillard et al., 2008). Still, theoretically, they cannot really be equally correct considering that they make opposite claims at a fundamental level. Although several studies have aimed to compare categorical and dimensional approaches to emotions (e.g., Eerola and Vuoskoski, 2011), the data reported have rarely any bearing on the fundamental assumptions of each approach^2^. The most important difference between a dimensional and a categorical approach is that the former assumes that emotions vary in a *continuous* manner in “emotion space,” whereas the latter assumes that there is *discontinuity* (discreteness) in “emotion space.” Though only few studies have directly addressed this essential aspect, those studies that have indicate that the continuity assumption is incorrect. That is, emotions show discreteness in terms of category boundaries, rather than continuity (Haslam, 1995).

In this article, we are concerned with emotional expression. Of particular importance in this context are studies which show that continuous variation in vocal emotion expressions is processed categorically (de Gelder and Vroomen, 1996; Laukka, 2005), since there are strong parallels among vocal and musical expressions of emotion (Juslin and Laukka, 2003; Table 7). If emotions conveyed in sound are not perceived in a continuous fashion, then other types of comparisons among the two approaches suddenly don't appear that important anymore—the dimensional approach has already been found wanting.

It's easy to see why categories are needed. Emotions function to guide decisions about future behavior. A continuous dimension of, say, *valence* is all very nice—but how are you going to use it? Exactly *how much is enough* to motivate a change in behavior? We need a “cut-off” or “stop rule” to make a decision; and once we have that—*voilà*—we have a *category boundary*. (In fact, even the traditional *pleasure* or *valence* dimension of the circumplex seems to imply a discrete boundary at some point—between positive and negative; approach and avoidance).

Categories are of crucial importance to human behavior: they aid inferences, communication, and decision making (cf. Markman and Rein, 2013). Hence, even Barrett (2006), a dimensional theorist of rang, appears to have accepted that “core affect” in terms of only two dimensions is insufficient to account for human emotions. She postulates a conceptual layer of categories of emotion on top of the two dimensions, and assumes that this layer is a social construction that mainly reflects language. But can emotion categories be dismissed so easily?

The argument that categorical perception of emotional expressions reflects language is partly based on findings that categorical perception involves a left-hemisphere bias in the brain. But Holmes and Wolff (2012) reported that categorical perception is not driven by language^3^ ; and emotion categories in vocal expression appear in other mammals, which obviously don't have a verbal language. For instance, the squirrel monkey has a limited number of vocal expression categories, which are associated with important events in the monkeys' life, such as warning calls (alarm peeps), threat calls (groaning), desire for social contact calls (isolation peeps) and companionship calls (cackling) (Ploog, 1992). Further, even in the first months of life, human infants are able to differentiate vocal expressions of emotions in infant-directed speech, and to respond adequately to their categorical messages (see Papoušek et al., 1990). While I certainly do not deny that language shapes several aspects of how we report—and perhaps even experience—emotions, it cannot fully account for the existence of discrete emotion categories. Panksepp (1998) have outlined distinct emotion systems, with neuroanatomical and neurochemical components in the mammal brain, associated with seven emotion categories. His emotion labels (with more commonly used labels within parentheses) are *seeking* (*interest*), *rage* (*anger*), *fear*, *lust* (*desire*), *care* (*tenderness*), *panic* (*sadness*), and *play* (*joy*). The point is that emotion categories go deeper than mere verbal labels in language.

While there seems to be a consensus today that dimensional approaches focus on subjective experience (*feelings*)—perhaps because they are so poorly able to account for other emotion components such as emotional expression—it is important to acknowledge that dimensional models did *not* derive from “raw data” of self-reported emotions. Instead, they were abstract dimensions that resulted from multivariate statistical techniques applied to similarity ratings of facial expressions and emotion labels (e.g., Plutchik, 1994). People do not spontaneously report emotions as coordinates within an abstract, multi-dimensional emotion space. Hence, dimensional models appear too reductionist. In the circumplex model, two emotions that are placed in the same position in the circular matrix may be very different. For example, *anger* and *fear* are two emotions that are highly correlated within this model because they are both high in arousal and unpleasantness. Yet they are very different in terms of their implications for the organism (Lazarus, 1991). Furthermore, musical expressions of the two emotions are quite different (see Juslin and Laukka, 2003; Table 7). This implies that the circumplex model cannot accommodate that we are able to distinguish *anger* and *fear* expressions.

Based on the above line of reasoning, I conclude that musical expression of emotion is likely to involve emotion categories, rather than mere dimensions. (As we shall see later, this does *not* preclude that there is an implicit dimensionality in emotion categories; cf. section Resistance Against Basic Emotions) If emotions tend to involve categories, then the next question is, which are those categories? Below, I suggest that an ecological perspective on emotions can be helpful to understand the kinds of categories that have been premiered throughout evolution. But the types of emotion that are expressed and recognized in music also reflect the precise process through which the emotional contents are transmitted.

## How does music express emotions? three types of coding

To explain why music appears to be expressing some emotions, rather than others, we need to take a closer look at the underlying process, particularly how the emotional meaning is *coded* in music (the specific manner in which the music carries the emotional meaning). I argue here that the emotional content of musical expression is constrained by the type of coding available and that distinct types of content are conveyed through different types of coding. Dowling and Harwood (1986) offered a useful categorization based on the ideas of Charles Pierce:

- *Icon* refers to a response based on formal similarity between the music and some other signal, such as vocal expression or human movement.
- *Symbol* refers to a response based on internal, syntactic relationships within the music itself.
- *Index* refers to a response due to an “arbitrary” association between the music and some other event or object.

These three principles have been referred to as “iconic,” “intrinsic,” and “associative” sources of musical expression, in an attempt to make the concepts easier to grasp (Sloboda and Juslin, 2001). In the following, I will consider these types of coding in music and their implications for the types of emotions expressed.

### Iconic coding: basic emotions

A first and very powerful source of perceived emotion in music reflects *iconic* coding. Juslin (1995, 1997, 1998, 2001) has repeatedly theorized that the code used in emotional expression in music performance is based on innate and universal “affect programs” for vocal expression of emotions. According to this “functionalist” framework—partly inspired by Spencer (1857)—the origin of iconically-coded expressions is to be sought in involuntary and emotion-specific physiological changes associated with emotional reactions, which strongly influence different aspects of voice production (for a review of the relationships among emotion, physiology and voice, see Juslin and Scherer, 2005). This notion was later named “Spencer's law” by Juslin and Laukka (2003). Because of its evolutionary origin, this is the type of coding that will have the most *uniform* impact on musical expression. I will show that iconically-coded expressions are intimately related to basic emotions.

#### The concept of basic emotions

The term *basic* or *discrete* emotions occurs frequently in the music psychology field today, typically to refer to certain emotions (*happiness, sadness, anger*, and *fear*), but without any deeper consideration of the theoretical basis of the concept. This is unfortunate, as it serves to obscure many of the issues under consideration.

First of all, it is quite possible to talk about emotions like *sadness, surprise, anger, happiness, interest*, and *fear* without adopting a basic-emotions perspective. Thus, simply adopting these emotions does not itself make one a “basic-emotion theorist.” (Otherwise, even Scherer would be a “basic-emotion theorist” because most of his studies have focused on these emotions; e.g., Scherer and Oshinsky, 1977; Banse and Scherer, 1996; Scherer et al., 2001). Hence, regardless of one's theoretical position, *sadness*, *happiness, anger, surprise*, and *fear* are obvious examples of emotions from “everyday life.” Therefore, my recommendation is to employ the term “basic emotion” only when one is embracing the theoretical basis of this concept, and to use the term “everyday emotions” when one is simply referring to emotions like *happiness, anger, surprise, fear*, and *sadness*, without wanting to commit to the underlying theory of basic emotions.

The concept of *basic emotions* refers to the idea that there is a limited number of innate and universal emotion categories, which are more biologically fundamental than others (Tomkins, 1962; Izard, 1977; Ekman, 1992; Oatley, 1992; Plutchik, 1994; Power and Dalgleish, 1997). Each basic emotion may be defined functionally in terms of a key appraisal of goal-relevant situations that have occurred frequently during evolution (e.g., Oatley, 1992). The situations include cooperation, conflict, separation, danger, reproduction, and caring. Support for basic emotions comes from a wide range of sources that include:

- Phylogenetic continuity of basic emotions (Plutchik, 1980)
- Early development of proposed basic emotions (Harris, 1989)
- Distinct brain substrates associated with basic emotions (Murphy et al., 2003)
- Distinct patterns of psychophysiological changes (Ekman et al., 1983)
- Cross-cultural accuracy in facial and vocal expression (Elfenbein and Ambady, 2002)
- Categorical perception of facial expressions of basic emotions (Etcoff and Magee, 1992)
- Clusters matching basic emotions in similarity ratings of affect terms (Shaver et al., 1987)
- Reduced reaction times in lexical-decision-tasks when priming words are taken from the same basic emotion category (Conway and Bekerian, 1987)

Not all of these sources of evidence are equally strong: thus, for example, the extent to which psychophysiological measures can distinguish among basic emotions is controversial, though recent multivariate approaches to emotion classification are promising (e.g., Kragel and LaBar, 2013). Yet, the most impressive evidence of basic emotions comes from studies of emotional communication (Juslin and Laukka, 2003).

#### Basic emotions in vocal and musical communication

To answer the question of which emotion categories we have, we first need to ask ourselves why we have categories *at all*; and, in particular, why we have emotion categories. Here, an ecological perspective on emotion could be helpful. Categories enable us to make important inferences (Corter and Gluck, 1992). For example, the ability to predict the probable behavior of another individual is quite useful: it allows the judge to adjust his or her behavior in order to affect the outcome of the interaction. Consequently I have argued elsewhere (Juslin, 1998) that when it comes to communication of emotion, the basic emotion categories represent the optimal compromise between two opposing goals of a perceiver: the desire to have the most informative categorization possible and the desire to have the categories be as discriminable as possible (Ross and Spalding, 1994). To be useful as guides to action, emotional expressions are typically decoded in terms of a few emotion categories related to important life problems such as danger (*fear*), competition (*anger*), loss (*sadness*), social cooperation (*happiness*), or caregiving (*love*) (Juslin, 2001).

In support, there is cross-cultural accuracy in decoding of basic emotions in vocal expression even in so-called traditional societies without any exposure to media (Bryan and Barrett, 2008). Critics of the basic-emotion approach in studies of vocal expression (Bachorowski, 1999) like to point out that it has been difficult to find distinct voice-profiles for basic emotions. Indeed, although basic emotions do present different acoustic features (Juslin and Laukka, 2003; Table 7), it's clear that the acoustic patterns obtained do not always neatly correspond to categories. But to look for discrete categories in the acoustic data is to look at the wrong place altogether. Categorical perception is a creation of the *mind*, it's not in the physical stimulus. The relevant support comes from work that shows that vocal emotion expression is *perceived* categorically (Laukka, 2005). The argument is that this evolved tendency to interpret emotional meaning in sounds in terms of certain categories places some constraints on musical expression also.

I have speculated (Juslin, 2001) that the origin of music lies in ceremonies of the distant past that related vocal emotion expression to singing: vocal expressions of basic emotions such as *happiness*, *sadness*, *anger* and *love* probably became gradually meshed with vocal music that accompanied associated cultural activities, such as festivities, funerals, wars, and caregiving. The implication is that basic emotions are “privileged,” in the sense that they are biologically prepared for effective communication.

That basic emotions are easier to convey reliably in musical expression is also partly an effect of the fact the communicative process involves partly redundant cues which limits the amount of information that may be conveyed through the “channel,” as captured by the *Lens Model* for music and emotion first proposed and implemented by Juslin (1995, 2000). This characteristic might also be explained in terms of evolutionary pressures: Ultimately, it is more important to avoid making serious mistakes (e.g., mistaking *anger* for *joy*), than to have the ability to make subtle discriminations among emotions (e.g., reliably recognizing different types of *joy*). Thus, a listener's interpretation of emotions in music will tend to gravitate toward basic categories.

#### Resistance against basic emotions

As shown above there are plenty of reasons to adopt a categorical approach in terms of basic emotions. Why, then, has the notion of basic emotions been treated with so much skepticism in the music field recently? The reasons may be different, depending on who the skeptics are. Among *musicians*, there may be a sense that the concept of basic emotions somehow implies a low level of musical sophistication. (Who would like to have his or her music compositions or performances described as “basic”?) As pointed out by Juslin and Lindström, (2010), however, the term *basic emotion* does not imply that the music itself is “basic”: indeed, “basic emotions may be expressed in the most sublime manner” (p. 356). The term simply highlights the fact that basic emotions are at the core of human emotions. (Moreover, for most theorists, the idea of basic emotions also means that there are more complex emotions; see section Beyond Basic Emotions: Intrinsic and Associative Coding) Yet, one source of resistance to basic emotions is probably the terminology as such.

One way to reduce resistance to the notion of basic emotions amongst musicians could be to demonstrate their natural relationships to the everyday praxis of musicians, even in classical music. Could it be the case that these terms used merely as shorthand for broad categories of emotion in musical expression in previous studies (Juslin, 2001) can be “translated” to some “language” more familiar to the working musician? Musical scores often include “expression marks” that serve to indicate not only the tempo of the music but also the intended expressive character of the music. In a recent study (Juslin and Wiik, submitted), professional performers and psychology students were required to rate a highly varied set of pieces of classical music with regard to 20 expression marks rated as common by music experts and 20 emotion terms rated as feasible in the context of musical expression (e.g., Lindström et al., 2003). When the ratings were combined, the analysis yielded highly significant correlations among expression marks and emotion terms—in particular for basic emotions (Table 2). The results may not be particularly surprising, given that expression marks typically involve reference to motion and emotion characters. But the point is that when music psychologists talk about basic emotions, they may well be referring to precisely the same expressive qualities that performers consider in expression marks throughout their daily work. Again we should not get too hung up on the superficial labels used to refer to the underlying emotion categories^4^.

**Table 2:** **Examples of correlations between commonly used expression marks in music scores and basic-emotion labels used by psychologists**.

**Expression mark**	**Emotion label**	**Correlation (*r*)**
*Dolce*	Tenderness	0.98^*^
*Espressivo*	Desire	0.85^*^
*Furioso*	Anger	0.92^*^
	Disgust	0.79^*^
*Grave*	Sadness	0.88^*^
*Scherzando*	Happiness	0.76^*^
*Spiritoso*	Surprise	0.94^*^
*Temoroso*	Anxiety	0.97^*^
	Fear	0.82^*^

* p < 0.01.

(based on Juslin and Wiik, submitted).

Among *music researchers*, resistance to basic emotions seems to be due to certain myths that have been allowed to flourish unchallenged, and that have contributed to a misunderstanding of the concept of basic emotions. Six of these myths warrant closer consideration here.

***Myth 1: “There is no agreement about which emotions are basic.”*** Basic emotions have been criticized, based on the fact that different emotion theorists have come up with different lists of emotions (Ortony and Turner, 1990). But this argument is, on reflection, a little suspect. There is a key question we should ask about the concept of basic emotions: does the concept help to narrow down and organize the field of emotion in a way that makes for greater agreement and consistency amongst those researchers who adopt the concept than amongst those who don't? If so, the concept is heuristic. Note that ideas about emotions depend crucially on how one *defines* an emotion. This helps to explain differences with respect to the lists of basic emotions proposed so far. How can we expect the authors to come up with the same set of basic emotions if they don't define emotions in the same way? The relevant question to ask is therefore: *is there agreement about which emotions are basic amongst those who define emotions in a similar way?* In fact, if we consider the authors who adopt similar definitions of emotions (e.g., in terms of their evolutionary adaptiveness), there is a lot of agreement about which emotions are basic (e.g., Plutchik, 1980). There is arguably more disagreement about the term “emotion” itself than about basic emotions (cf. Kleinginna and Kleinginna, 1981). Yet, few would argue that we should abandon the term “emotion.”

***Myth 2: “Basic-emotions are incompatible with appraisal theory.”*** Sometimes the basic-emotion approach is contrasted with “appraisal theories” (Scherer, 1984), which aim to describe the processes through which an emotion is aroused. This is misleading, as it implies that the basic-emotion approach is somehow incompatible with appraisal. In fact, it turns out that many appraisal theorists embrace the notion of basic or primary emotions (see Lazarus, 1991; Roseman, 1991; Stein and Trabasso, 1992). Appraisal is a fundamental aspect of emotion induction that must be part of *any* emotion theory regardless of how it conceptualizes the resulting emotions. A component-process theory (e.g., Scherer, 1984) does not differ from a basic-emotion theory because it involves appraisal: The primary difference between the two types of theories is that the former assumes that there are as many emotion categories as there are possible outcome combinations of the appraisal-criteria included. (To my knowledge, this essential assumption has never actually been tested and verified by any researcher). The latter type, in contrast, assumes that cognitive appraisals typically result in a fewer number of broad categories, with more differentiated appraisals producing nuances *within* the categories, rather than additional categories. Regardless, basic-emotion theories are compatible with attempts to model the appraisal process that produces an emotion^5^.

***Myth 3: “Basic emotions are crude and lacking in nuance.”*** This refers to the common view that emotion categories do not allow for the occurrence of subtle nuances within a category. This reflects a misunderstanding of the very concept of a category. Just as there are different shades of *blue*, there can be different shades of *sadness*. The notion of basic emotions implies that, emotions from distinct basic-level categories are more different from one another than are different emotions from within the same category (e.g., *sadness* and *joy* differ more than, say, *sadness* and *melancholy*); this doesn't preclude that there are nuances *within* categories as well. The notion of an emotion category is nicely captured by the word “emotion family” (e.g., Ekman, 1992). Each family includes a “theme” and its “variations.” The “theme” represents the common characteristics of the basic emotion and the “variations” all the subtle nuances and shadings that might occur *within* the category. Laukka and Juslin (2007) reported that listeners could accurately recognize various intensity levels (high or low) of basic emotions in both vocal and musical expressions. Hence, there's an implicit dimensionality within basic-level emotion categories. Schubert (2010) points out that although we often think of using continuous-response methodology only with respect to dimensional models, it's perfectly possible to collect continuous ratings of discrete emotions also (e.g., to rate the amount of *sadness* while the music unfolds). In addition, many emotion researchers postulate “secondary” or “mixed” emotions which are founded on basic emotions, but that involve “blends” of emotions (Plutchik, 1994), or specific cognitive appraisals which occur together with a basic emotion (Oatley, 1992). Hence, Johnson-Laird and Oatley (1989) were able to sort several hundreds of emotion terms into just five basic emotion categories or some subset of them. Basic-emotion theories are able to accommodate diversity and nuances, including ebb and flow in emotion over time.

**Figure 1:**
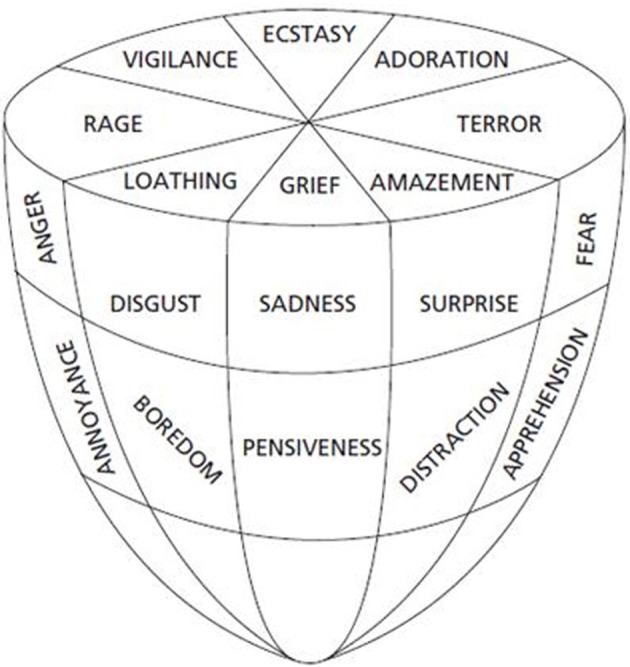
**Plutchik's “cone model” of emotion (adapted from Plutchik, 1994)**.

***Myth 4: “Basic emotions are always full-blown responses.”*** Basic emotions are commonly depicted by critics in a stereotyped manner, which borders on caricature: it's usually about hair-raising fear when confronted by a bear! But basic emotions may vary in intensity (e.g., from *frustration* or *irritation* to *anger* and *rage*). There is nothing in the concept of basic emotions as such that requires that the emotion will always be intense. Basic emotions are typically portrayed in such a way by critics in order to make the emotions appear irrelevant in everyday life (or in music). Are basic emotions relevant in everyday life? In the context of vocal expression, Cowie et al. (1999) asked participants to select a subset of emotions that they thought were important in everyday life. This produced a list of 16 emotions and labels chosen included basic emotions in different variants such as *anger, fear, happiness, sadness, love, worry, interest* and *affection* (cf. Panksepp's seven emotional systems): we feel *irritated* when we can't find a parking space; *tender* when our children greet us; *anxious* when we receive letters from the tax office; or *enthusiastic* when we get a paper accepted. The mere fact that most emotions experienced in everyday life aren't particularly intense does not imply that they do not involve basic-emotion categories^6^. Consider Plutchik's (1994) *cone model* of basic emotions (Figure 1). The circular arrangement shows the degree of similarity among the emotions, whereas the vertical dimension shows the intensity dimension. One consequence of this arrangement is that emotions of a lower intensity are closer to each other, and hence more similar, than are emotions of a high intensity. It may be that music often operates in the lower section of the cone, rather than in the extreme section representing “full-blown emotions,” but the same emotion categories are still involved. Therefore, we may not always “detect” discrete emotions in everyday life situations or in musical expressions, simply because milder versions of basic emotions involve more subtle differences.

***Myth 5: “Basic emotions are not relevant in music.”*** The above myths can explain a further myth: that basic emotions are irrelevant in the context of musical expression. One moment's reflection suggests the opposite—if there is *any* type of emotions that could be expected to have a strong and natural link to musical expression, then it's the basic-emotion type: basic emotions can be conveyed nonverbally through gesture and tone of voice using similar patterns (e.g., Clynes, 1977; Juslin, 1997), whereas more complex emotions don't have similarly distinct nonverbal patterns. We also saw that emotions that are regarded as basic emotions (e.g., *happiness, sadness, anger, tenderness, fear*) seem easiest to express and perceive in music, as indexed by listener agreement (Gabrielsson and Juslin, 2003) and ratings by both musicians (Lindström et al., 2003) and listeners (Juslin and Laukka, 2004). Zentner and Eerola (2010) submit that discrete-emotion models were not developed to study music. This is of course true, but in the context of perceived emotion, this misses the greater point: that music probably evolved on the foundation of vocal expressions of basic emotions. Hence, examples of such basic emotions may easily be found also in commercially available recorded music. For example, Leech-Wilkinson (2006) offers a large number of examples of “expressive gestures” used by singers to express basic emotions, such as *fear, sadness, anger, love*, and *disgust* in Schubert Lieder (see also analysis by Spitzer, 2010). Further, if we leave classical music aside for the moment—since it is a minority interest in the world, and even in the Western world (Hargreaves, 1986)—and look at the types of music most frequently heard in everyday life, we find that popular music involves songs about things that matter to people, the stuff that makes them happy, sad, angry, afraid, or tender.

***Myth 6: “Basic emotions have dominated in studies of music and emotion.”*** This concerns the increasingly common claim that basic or discrete emotions have somehow dominated in music and emotion research. The actual data reveal something else. Eerola and Vuoskoski (2013) recently reviewed studies of music and emotion published over a ten-year period (from 1988 to 2009). They found that about one third of these studies adopted a basic or discrete-emotions perspective. This shows, then, that the majority of studies of music and emotion have *not* focused on basic emotions. This is even more true, if one extends the time-frame of the overview. For instance, Gabrielsson and Juslin (2003), who reviewed studies of emotional expression in music from the 1890's, observed that the concept of basic emotions, and other influences from emotion psychology in general, have come into studies of musical expression quite recently, and then primarily in studies of music performance. In most of the investigations to date, the emotions measured have instead been chosen based on statements from philosophers and music theorists; suggestions from previous studies; and intuition, folk psychology, and personal experience. All together, the emotion labels used in previous work are counted in hundreds. Therefore, the view that basic emotions have dominated in previous studies of music and emotion is largely a “straw man”^7^.

#### A positive explanatory role of basic emotions

If we can get past the above myths about basic emotions, and consider the concept on its own merits, we may find that it can be highly heuristic to our understanding of musical expression. Few researchers in the music field have explicitly adopted a basic-emotions approach (but see Clynes, 1977). I proposed such an approach specifically in the context of studies of emotional expression in the *performance* of music (and not as an all-encompassing solution for the field of musical emotion), because I thought the concept could uniquely help to account for several of the findings in that field (see Juslin, 1997). The findings that have amassed since then have only reinforced this belief. Hence, consistent with the idea that emotional expression in music performance is mainly based on a code for vocal expression of basic emotions that has served important functions throughout evolution is evidence that:

- Basic emotions in vocal expressions can be recognized cross-culturally, even in traditional cultures (Bryan and Barrett, 2008)
- Basic emotions in vocal expression are perceived categorically (e.g., de Gelder and Vroomen, 1996; Laukka, 2005)
- It is notoriously difficult to “retrain” a participant so as to express a specific basic emotion with a different expressive pattern (Clynes, 1977, pp. 44–45)
- There are significant similarities between vocal expression and musical expression of basic emotions (Juslin and Laukka, 2003; Table 7)
- There is a similar pattern of age-related differences in recognition of emotions from vocal expression and music performance (Laukka and Juslin, 2007; see also Lima and Castro, 2011)
- Congenitally *amusic* individuals (with deficits in processing acoustic and structural attributes of music) are significantly worse than matched controls at decoding basic emotions in vocal expressions (Thompson et al., 2012)
- Basic emotions are easier to communicate than complex emotions in music (Gabrielsson and Juslin, 1996; cf. Senju and Ohgushi, 1987)
- Basic emotions in music can be recognized cross-culturally (Fritz et al., 2009)
- Basic emotions in music show high cross-cultural agreement, whereas non-basic emotions show low cross-cultural agreement (Laukka et al., 2013)
- Basic emotions such as *sorrow, anger, love, joy* and *fear* are explicitly part of many non-Western theories of musical emotions (e.g., Becker, 2004, p. 58)
- Decoding of basic emotions in music is very quick (Peretz et al., 1998; Bigand et al., 2005)
- Decoding of basic emotions in music does not require musical training (e.g., Juslin, 1997; Vieillard et al., 2008)
- Expression of basic emotions in music does not require musical training (Yamasaki, 2002)
- Even children (3 or 4 years old) are able to decode basic emotions in music with better than chance accuracy (Cunningham and Sterling, 1988; Terwogt and van Grinsven, 1991)
- Even children are be able to use voice-related cues to express basic emotions in their songs (Adachi and Trehub, 1998)
- The ability to decode basic emotions in music performances is correlated with measures of emotional intelligence (Resnicow et al., 2004)
- There are cross-cultural similarities in cue utilization for features shared between vocal expression and musical expression (Balkwill and Thompson, 1999; Laukka et al., 2013)
- Decoding of basic emotions in music performances involves many of the same brain regions as perception of basic emotions in vocal expression (Escoffier et al., 2013)

It is my strong belief that no other emotion approach can nearly as convincingly account for the above findings regarding expression of emotion in music performance. The dimensional approach would have to explain why there is categorical perception of emotional expression if emotions are processed as continuous dimensions. It would also have to explain why some emotions are more easily expressed and recognized than others, if all emotions can be placed along the same continuous dimensions. Component-process theories would have to show that there are as many recognizable emotion categories in musical expression as there are possible appraisal-combination outcomes. This is a tall order, and I do not expect it to happen anytime soon. In contrast, a basic-emotions approach (Juslin, 1998) *predicts* categorical perception of emotions and higher listener agreement or decoding accuracy for emotions such as *happiness, sadness, anger, fear*, and *tenderness*.

### Beyond basic emotions: intrinsic and associative coding

The idea that basic emotions are “privileged” in musical expression does *not* imply, however, that other emotions cannot be conveyed in music also. It seems possible for music to convey more complex emotions under certain circumstances, even though there will tend to be lower agreement between listeners for such emotions (Senju and Ohgushi, 1987; Laukka et al., 2013). Part of the reason for this tendency is that more complex emotions are coded differently: they involve intrinsic and associative coding.

#### Intrinsic coding

Intrinsic coding involves internal syntactic relationships within the music itself. Music theory involves frequent references to tonal or harmonic motion (Lerdahl and Krumhansl, 2007), even gravitational forces between tones and chords (Larson and Van Handel, 2005), which can create “tension,” “release,” “climax,” “repose,” and “relaxation.” Although Meyer's (1956) well-known theory focused primarily on how the thwarting of musical expectations might *arouse* emotion in listeners, it seems likely that this internal play within the musical structure could also affect *perceived* emotions (e.g., the emotional intensity; see Sloboda and Lehmann, 2001; Timmers and Ashley, 2007, for examples). Intrinsic sources of musical expression in music have rarely been investigated thus far, but they are unlikely to express specific emotions by themselves. Rather, their signification appears quite broad and mainly helps to qualify specific emotions conveyed by iconic or associative coding. By contributing dynamically shifting levels of tension, arousal and stability, they may help to express more complex, time-dependent emotions, such as *relief* and *hope*. This type of coding may require longer music excerpts in order to be truly effective, while most studies to date have used relatively short excerpts (Eerola and Vuoskoski, 2013).

#### Associative coding

Finally, music might also be perceived as expressive of emotions through *associative* coding. In other words, a performance of music may be perceived as expressive of a specific emotion simply because something in the music (a melody, timbre) has been repeatedly and arbitrarily paired with other meaningful stimuli or events in the past. Organ music could be perceived as expressive of “solemnity” or “spirituality,” simply because it has been heard often in churches. Dowling and Harwood (1986) offered a classic example, in terms of Puccini's use of the first phrase of the “Star Spangled Banner” in *Madame Butterfly* to signify a feeling of “patriotism.” Associative coding plays a crucial part in Wagner's *Leitmotif* strategy, where specific melodic themes are associated with particular characters in the drama. Included in this coding subtype are also expressive meanings which are purely conventional. Throughout music history, there are several examples of systems for emotional communication primarily based on convention (e.g., “the doctrine of affections”; see Buelow, 1983). Through this type of coding, music may achieve a more precise and complex expression, but its recognition will depend on having the necessary knowledge or experience. Hence, emotional expression through this type of coding will necessarily be less cross-culturally invariant and more context and/or listener dependent. Beyond a certain level, the associations will be deeply personal. DeNora (2001) describes the case of “Lucy,” whose hearing of the Schubert “Impromptus” brings connotations of “comfort” because her father used to play these pieces when she was falling asleep after dinner.

Cross (2012) notes that music appears to be “a strangely malleable and flexible phenomenon” (p. 265), in that “one and the same piece can bear quite different meanings for performer and listener, or for two different listeners” (p. 266). Some of this so-called *floating intentionality* or “aboutness” (Cross, 2012, p. 266) is perhaps beyond systematic modeling, but may still be explored in terms of in-depth interviews and music analysis. For example, Delis et al. (1978) suggest that listeners construct a story in relation to the music, in order to better remember it. Moreover, some listeners perceive the music to reflect their own personality (how they think and feel), thereby confirming their self-identity (Gabrielsson and Lindström Wik, 2003). Music analyses in the more “hermeneutic” tradition may also be concerned with associative codings: for instance, when Hatten (1994) analyses the *Cavatina* of Beethoven's string quartet op. 130 and notes that “the ‘willed’ (basically stepwise) ascent takes on a *hopeful* character supported by the stepwise bass … ” (p. 213, italics added), there is no doubt that this is how Hatten hears the music; there is also little doubt that few other listeners would hear the piece in exactly the same way (unless, perhaps, they have read Hatten's persuasive interpretation).

The real powers of intrinsic and associative sources of perceived emotions in music might lie in their ability to modulate or extend the expression provided by iconically-coded sources, as discussed in the following section.

#### Codings combined: multiple layers of musical expression

**Figure 2:**
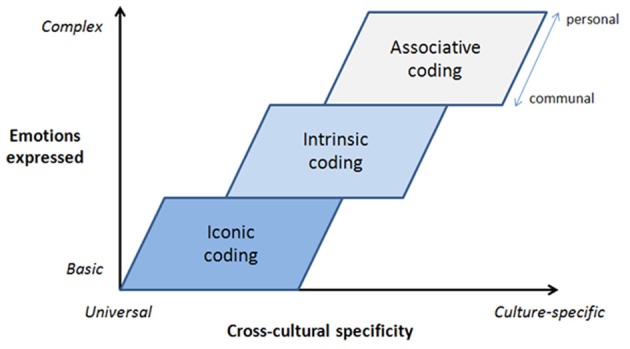
**Multiple-layer conceptualization of musical expression of emotions**.

Figure 2 illustrates the conceptualization of musical expression proposed in this article: there are three primary types of coding which correspond to three “layers” of musical expression of emotions. The bottom (“core”) level is constituted by iconically-coded basic emotions (based on vocal expression). This layer may explain universal recognition of basic emotions, in both vocal (Bryan and Barrett, 2008) and musical (Fritz et al., 2009) expression. However, this layer can be extended, qualified, or even modified by two additional layers in terms of intrinsic and associative coding, enabling listeners to perceive more complex emotions, which however are less cross-culturally invariant. Intrinsically-coded expression may add dynamically changing contours (e.g., variation in “tension,” “arousal,” or “intensity”) which help to shape more time-dependent emotional expressions (e.g., conveying “relief” may depend on changes over time). Associative coding adds an even richer level of complex emotions, although typically with a low level of cross-cultural or even inter-individual agreement. This layer can furthermore be divided into a more “communal” associative subsection/dimension and a more “idiosyncratic” (or deeply personal) subsection/dimension. The “communal” subsection involves the common associations of a particular social group, as constituted by shared experiences (group identity) or musical conventions. At the final layer of expression, the idiosyncratic layer, a listener can perceive just about *any* emotion in the music, through deeply personal associations.

Conceived in this manner, it is easy to see how perception of emotional expression in music might lead to *both* agreement (Juslin, 1997) and disagreement (Huber, 1923); cross-cultural similarities (Fritz et al., 2009) and differences (Gregory and Varney, 1996); a shared meaning (Sloboda and Juslin, 2001, p. 95) and deeply personal meaning (Gabrielsson and Lindström Wik, 2003), sometimes, perhaps, even within the same study or piece of music. Further, one might conceive of “mixed emotions,” resulting from different emotional meaning at different layers, somewhat akin to what Cohen (2001) refers to as “emotional polyphony” (p. 252).

This multiple layer notion of musical expression might account for some previous findings. For example, Brown (1981) studied music listeners' ability to recognize emotions in pieces from different styles and genres in classical music. He chose 12 musical excerpts and asked listeners, both musicians and non-musicians, to sort them into six broad emotion categories. In a second task, listeners were instead required to identify six pairs out of 12 other musical excerpts representing “Variations on Sadness” (i.e., variants *within* the same broad emotion category). While listeners were quite successful in the first task, they were not in the second task, until Brown supplied his own descriptions of the six sadness categories. However, non-musicians were still unsuccessful. Brown thus concluded that if the different expressions are not too similar (as in the first task), the emotion categories can be identified even by persons not highly knowledgeable about classical music; however, with pieces as close in expression as in the variations on sadness “the agreement on synonymous pairs can only be achieved by listeners highly conversant with the traditions involved” (p. 264). One may re-interpret these results as follows: the recognition of broad basic emotion categories was based on iconically coded expression which does not require musical expertise; the recognition of more complex or subtle nuances within the categories was based on associative coding which requires some knowledge of musical conventions.

Similarly, in a recent cross-cultural study of musical expression of emotions by Laukka et al. (2013), it was found that decoding of basic emotions was rather robust regardless of whether the music was familiar or not—presumably because it's based on the core layer of iconically coded expression. In contrast, decoding of non-basic emotions was more limited as it merely occurred for some listener groups and/or for familiar musical cultures. These emotions were probably based to a greater extent on associative coding (e.g., social conventions) at the third layer of expression (see Figure 2).

The relative importance of the three layers of musical expression could vary as a function of musical genre, historical context, as well as various listener characteristics. Still, I think that iconic sources tend to be the most powerful—because associative sources are too individual and intrinsic sources are too indeterminate. Hence, iconic sources, linked to basic emotions, account for the lion's share of musical expression. These sources have a clear cross-cultural component due to their direct link to autonomic arousal and the human voice. Consequently, it does not appear far-fetched to assume that the so-called “psychophysical cues” in Balkwill and Thompson's (1999) *cue redundancy model* mainly correspond to iconically-coded basic emotions that can be cross-culturally recognized, whereas their “culture-specific cues” partly correspond to emotions coded more in terms of associative or intrinsic sources. Similarly, a decomposition into different types of coding might help to account for both similarities and differences between vocal expression (e.g., Juslin and Scherer, 2005) and musical expression (Gabrielsson and Juslin, 2003): Iconic coding of basic emotions will tend to be similar across the two channels (Juslin and Laukka, 2003), but associative and intrinsic sources of emotions will diverge, since their different functions in human life will shape conventions underlying their use differently.

## The special case of arousal of felt emotions

So far, this paper has been concerned exclusively with expression and perception of emotions. However, most researchers believe that music can also *arouse* felt emotions in listeners under certain circumstances. This issue is not uncontroversial (Juslin and Västfjäll, 2008), although as eloquently put by Ball (2010), “no one can doubt that some music is capable of exciting some emotion in some people some of the time” (p. 257). It is very important to distinguish arousal of emotions from expression and perception of emotions, because the emotions involved may be different depending on the process (Juslin and Laukka, 2004).

To be clear, the present author has *never* suggested that arousal of felt emotion during music listening is limited to basic emotions; quite the contrary, for over a decade I have repeatedly observed that music arouses a wide range of emotions (Juslin and Laukka, 2004; Juslin, 2005, 2011, 2013; Juslin and Västfjäll, 2008; Juslin et al., 2011). The notion that basic emotions are “privileged” applies *only* to expression and perception of emotion, not to arousal of emotion (And even in the case of expression and perception, I have allowed for, and examined, more “complex” emotions; e.g., Juslin et al., 2004). Despite this, it's not uncommon for scholars to give the impression that my position is, or has been, that music arouses only basic emotions. Again, it is helpful to consider the actual findings of relevance to the issue at hand.

Survey studies of the prevalence of musical emotions suggest that music arouses quite a wide range of states. Among the most frequently reported emotions to date are the following broad categories: *Calm-relaxation, happiness-joy, nostalgia-longing, interest-expectancy, pleasure-enjoyment, sadness-melancholy, arousal-energy, love-tenderness, pride-confidence* as well as various synonymous terms (Wells and Hakanen, 1991; Sloboda, 1992; Juslin and Laukka, 2004; Juslin et al., 2008, 2011; Zentner et al., 2008). “Mixed” emotions (e.g., both *joy* and *sadness*) also occur—but in a minority of the events (13% in Gabrielsson, 2010; 11% in Juslin et al., 2011).

Hence, previous findings indicate that the emotions aroused by music include basic emotions, but also include many other emotions, depending on which underlying mechanism caused the emotion (see Juslin, 2013, for further discussion). Even the supposedly “music-specific” scale for measuring emotional reactions to music, GEMS (e.g., Zentner et al., 2008), includes basic emotions (e.g., *sad* → SADNESS; *irritated* → TENSION; *in love* → TENDERNESS; *joyful* → JOYFUL ACTIVATION). Thus, Lamont and Eerola (2011) suggest that GEMS “contains significant redundancy in comparison to traditional models” (p. 142). We need to identify the real points of agreement and disagreement: researchers agree that music arouses a wide range of emotions that go beyond basic emotions. They agree that music arouses more positive than negative emotions. They do *not* agree, however, that there exist unique emotions aroused when and only when people listen to music (Juslin, 2013).

## Conclusion: ending the basic-emotion bashing

Let us return to the main question posed at the outset of this article: What does music express? Or, formulated more precisely: What are the emotional contents that listeners may perceive in music? As noted at the beginning, the question may have different answers depending on how we operationalize the notion of expression: Is it sufficient that any single listener perceives an emotion? Or should there be a minimum level of listener agreement? Or should the perceived emotion correspond to what the composer intended?

These various senses in which music can be said to express emotions are largely integrated in the present approach, which may be summarized as follows: There are three distinct layers of perceived musical expression of emotions. Each layer corresponds to a specific type of coding of emotional meaning. The “core” layer is constituted by iconically-coded basic emotions that can explain recent findings of universal recognition of basic emotions in vocal expression and music. The “core” layer can be extended, qualified and sometimes even modified by additional layers in terms of intrinsic and associative coding, which enable listeners to perceive complex emotions. These additional layers of expression are less cross-culturally invariant, though, and more dependent on the social context and/or the individual listener. At the “core” level of basic emotions, vocal and musical expression are fairly similar. At the additional layers that involve more complex emotions, vocal and musical expression begin to diverge from one another, due to the unique functions and uses associated with each modality. Depending on how expression is coded in particular pieces of music, we may expect to find different results across empirical investigations. Hence, I have argued that one might easily obtain evidence of either cross-cultural invariance or diversity, simply depending on how one is selecting the music in studies (Juslin, 2012).

Research to date has primarily focused on iconically-coded expression of emotions in music. It would thus be interesting to explore in future studies how associative and intrinsic sources contribute to expression, *beyond* basic emotions produced by iconically-coded sources. Still, while there is more to expression in music that basic emotions, as I have tried to show, basic emotions remain at the core of the process, and cannot be ignored. An approach that focuses only on basic emotions presents an incomplete picture (see Figure 2), while an approach that ignores basic emotions is plainly inadequate. Recent critiques of the basic-emotion approach in the music field have been marked by myths and misunderstandings (or by hidden agendas). Empirical data, in contrast, illustrate the value of the concept of basic emotions in accounting for musical expression of emotions (section A Positive Explanatory Role of Basic Emotions).

Hence, in closing this essay, I would like to call for an end to the “basic-emotion bashing,” in an attempt to offer a more nuanced view. As I have tried to show, a distinction between basic and complex emotions, and its link to various types of coding, can help to account for several findings concerning musical expression of emotions. The basic emotions represent the crucial link between our ancient past and modern music making, and are part of the reason that music is sometimes, perhaps justifiably so, called a universal language of the emotions.

### Conflict of interest statement

The author declares that the research was conducted in the absence of any commercial or financial relationships that could be construed as a potential conflict of interest.

## Footnotes

^1^Notably, the nine emotion clusters in Schubert's (2003) “updated” version of Hevner's (1936) adjective clock are quite similar to the most highly ranked emotion terms shown in Table 1.

^2^Eerola and Vuoskoski (2011) noted that although both discrete-emotions and dimensional ratings of perceived emotions in music showed a high internal consistency, the discrete-emotions model showed a somewhat lower consistency for ambiguous examples of emotions (poor emotion examplars). However, the dimensional model performs better mainly because it ‘by-passes’ the task of allocating perceived emotions to an emotion category.

^3^Bigand et al. (2005) also reported categorical effects that are unlikely to be a by-product of linguistic labelling, in the context of emotion judgments of music excerpts (p. 1130).

^4^Some musicians resist description of music in words more generally, on the basis that language is incapable of capturing all the musical nuances, but this limitation obviously applies to all emotion approaches discussed here.

^5^However, recall that emotions can be aroused in many different ways (Izard, 1993; Juslin, 2013). Hence, there lies a danger in defining emotion categories solely on the basis of specific appraisal outcomes.

^6^Admittedly, many researchers prefer to look at intense emotions, but that's because it may be easier to detect *effects* of emotional responses on various measures if one uses emotion episodes with a high intensity.

^7^One exception is a set of studies of *performance*, which used the so-called “standard paradigm” to investigate whether musicians can communicate various basic emotions to listeners (reviewed by Juslin and Laukka, 2003).
